# Structural and Thermoelectric Properties of Cu Substituted Type I Clathrates Ba_8_Cu_x_Si_~32−x_Ga_~14_

**DOI:** 10.3390/ma12020237

**Published:** 2019-01-11

**Authors:** Yue Dong, Xueyong Ding, Xinlin Yan, Long Zhang, Tianhua Ju, Chenghong Liu, Peter Rogl, Silke Paschen

**Affiliations:** 1 School of Metallurgy, Northeastern University, Shenyang 110819, China; dong_y2014@163.com (Y.D.); jthua89@gmail.com (T.J.); hcl123love@126.com (C.L.); 2 Institute of Solid State Physics, Vienna University of Technology, Wiedner Hauptstr. 8-10, 1040 Vienna, Austria; paschen@ifp.tuwien.ac.at; 3 State Key Laboratory of Metastable Materials Science and Technology, Yanshan University, Qinhuangdao 066004, China; lzhang@ysu.edu.cn; 4 Institute of Materials Chemistry, University of Vienna, Währingerstr. 42, 1090 Vienna, Austria; peter.franz.rogl@univie.ac.at

**Keywords:** thermoelectric materials, type I clathrate, single crystal growth, single crystal X-ray diffraction

## Abstract

With an attempt to improve the thermoelectric properties of type I clathrates in the Ba-Ga-Si system, we introduce Cu into the framework of the crystal structure. Single crystals are prepared in Ga-flux and characterized by X-ray diffraction techniques and transport measurements for the structural and thermoelectric properties. Our composition analyses show that only a small amount of Cu is determined in the clathrates. The single crystal X-ray diffraction data refinements confirm that Ga atoms prefer the 6*c* and 24*k* sites and avoid the 16*i* sites in the crystal structure. The small amount of Cu affects the crystal structure by compressing the tetrakaidecahedral cage along the direction perpendicular to the six-atom-ring plane. This could be the reason for the high charge carrier concentration, and low electrical resistivity and Seebeck coefficient. We analyze the principal mechanism for our observation and conclude that the Cu substitution can adjust some subtle details of the structure, maintaining the Zintl rule in the type I clathrates.

## 1. Introduction

As thermoelectric (TE) materials directly convert thermal energy to electric energy or vice versa, they are promising for applications in refrigeration and energy recovery devices [1,2]. The currently low energy conversion efficiency, however, restricts the application ranges. Efforts to improve the conversion efficiency have never stopped. The conversion efficiency of TE devices increases with *ZT*, the dimensionless figure of merit, which is defined as *ZT* = *S*^2^*T*/*ρ*(*κ_e_* + *κ_ph_*) [1,3], where *S* is the Seebeck coefficient (or thermal power), *ρ* the electrical resistivity, *T* the absolute temperature, *κ_e_* and *κ_ph_* the electronic and phonon contributions to the total thermal conductivity *κ*, respectively. To pursue high *ZT*, new technologies, new materials, new theories, and new concepts have been applied and discovered in materials research in the past decades which doubled or tripled *ZT* values reached in conventional material systems. One of the famous concepts is the so-called “phonon glass, electron single crystal” (PGEC), initiated by Slack [4], which suggested that compounds with amorphous-like thermal conductivity and crystal-like electrical conductivity should be promising as TE materials. This concept has orientated the research towards materials with large atomic thermal displacement parameters of heavy atoms in some crystallographic positions, especially cage-like compounds. 

**Figure 1:**
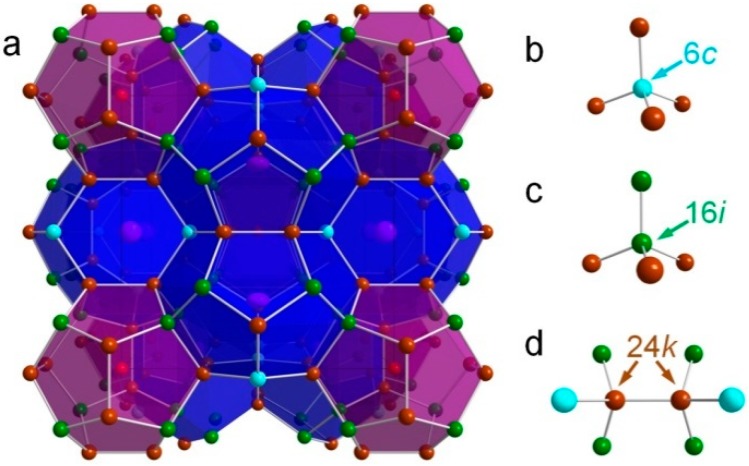
Crystal structure of type-I clathrate (**a**) and atomic environment (tetrahedral bonds) in different sites (**b**) 6*c*; (**c**) 16*i*; and (**d**) 24*k*.

Type I inorganic clathrates are one of the cage compounds that have been extensively studied for their crystal structure and TE properties. Type I clathrates, which are considered as particularly interesting PGEC candidates [5], have a guest–framework structure, where the framework is formed by IVA elements (Si, Ge, Sn) and the guest atoms come from alkaline metal, alkaline–earth metal, halogen, or rare earth elements. The framework consists of two different cages, one containing 20 atoms (small dodecahedra), and the other 24 atoms (tetrakaidecahedra) (Figure 1). The crystal structure is formed by face sharing polyhedra: two dodecahedra and six tetrakaidecahedra and inserting guest atoms into these cages, altogether 54 atoms per unit cell yielding a formula A_8_X_46_ (A is the guest atom and X is the framework atom). Type I clathrates typically have low thermal conductivity [6,7] due to (1) guest atom rattling because of its weak bonds to the framework atoms, which adds resonant scattering for phonons, and (2) low phonon speed because of a flat phonon dispersion associated with the framework topology. On the other hand, type I clathrates fulfill valence balances based on the Zintl concept [8], which tells that the framework atoms accept electrons donated by the guest atoms, forming the necessary electronic configurations for atomic bonds. The valence balanced compounds are of semiconducting character and thus have a large Seebeck coefficient, which is a prerequisite for materials of high TE performance.

Guided by the Zintl concept, substitution with transition metal (TM) elements and/or IIIA elements for IVA elements in the framework is usually effective to optimize the charge carrier concentration [8,9,10]. A series of compounds have been investigated [11,12,13,14,15,16,17,18,19,20,21,22,23,24,25,26,27,28,29] and a high value of *ZT* = 1.35 has been achieved at 900 K for Ba_8_Ga_16_Ge_30_ [30], which inspired more enthusiasm to pursue higher *ZT* in similar clathrates. The iso-structural Ba_8_Ga_16_Si_30_ clathrate has also attracted interest and has been studied by several researchers [31,32,33,34,35,36,37,38,39,40,41,42,43,44,45,46,47]. Eisenman et al. was first to provide the structure details for this clathrate [31]. Bentien et al. reported the Ga site preferences in the host structure [41,42]. Semiconducting behavior due to the vacancies in the framework sites was reported by Nataraj et al. [37,38,39]. A maximum *ZT* value of 0.75 at 1000 K was reached with a high Ga content 17.13 at./f.u. (atom per formula unit) in the work of Deng et al. [43]. Anno et al. [44,45,46] studied the solid solubility of Ga in Ba_8_Ga_x_Si_46−x_ and found the maximum value x = 14.8. The largest *ZT* value of 0.8 at 900 K can be reached with a carrier concentration of about 2 × 10^20^ cm^−3^. These studies indicate that it is very difficult to reach the Zintl composition, where the Ga content should be exactly 16 at./f.u.. Deviation from the Zintl composition usually results in vacancies in the crystal structure, which increases the difficulty for the prediction of the charge carrier concentration by the Zintl rule for the optimized charge carrier concentration. Introducing lower valence elements could be one of the solutions, by taking advantage of the valence difference between these elements (such as TM elements) and Ga. Attempts have been pursued with the substitution of Zn and Cu elements for the framework atoms [48,49,50].

Stimulated by such ideas, in the present work, we attempted to study the influence of Cu substitution for the framework atoms on the structural and thermoelectric properties. To omit grain boundary effects on the composition and the intrinsic transport property evaluation, we grew single crystals with Ga self-flux. We found that the Cu content in the crystals is very small. However, structural analyses showed that even a small amount of Cu affects the crystal structure and TE properties. We further compared our results with the literature data, mainly focusing on Cu-free compounds where the Si content is similar [31,35,38,39,42,46,50].

## 2. Materials and Methods

Aiming at preparing crystals with composition of Ba_8_Cu_2_Si_30_Ga_14_, pure elements (total 1.5 g) were weighted in a stoichiometric ratio. With additional Ga (with a flux to sample volume ratio of 5:95), the elements were put into an Al_2_O_3_ crucible which was sealed in a quartz tube in vacuum of 10^−5^ Pa to avoid possible oxidation at high temperatures. The tube was heated from room temperature to 1100 °C within 6 hours in a temperature-controlled furnace. After 3 h, the tube was cooled down to 800 °C with a cooling rate of 3 °C/h and kept at this temperature for 2 days. The remaining Ga was then decanted, and the crystals were separated by centrifugation. 

The quality of as-grown single crystals was checked by Laue measurements (COS, Micro Photonics Inc., Allentown, PA, USA), X-ray powder diffraction (XRD, X’Pert PRO, PANalytical B.V., Almelo, The Netherlands), and polarization microscopy (Eclipse LV100 POL, Nikon Instruments Inc., Melville, NY, USA). The composition of each single crystal was determined by energy dispersive x-ray spectroscopy (EDX, probe size: 1 μm) with a scanning electron microscope (SEM, Zeiss Supra 55VP, Carl Zeiss AG, Oberkochen, Germany) operated at 20 kV. For two selected single crystals, several points from different parts were measured and an average composition was obtained. 

For crystal structure investigation, small single crystals (50~80 μm) were extracted from mechanically crushed large crystals. Inspection on an AXS-GADDS texture goniometer (Bruker AXS GmbH, Karlsruhe, Germany) assured high crystal quality, and provided unit cell dimensions and Laue symmetry of the specimens prior to an X-ray intensity data collection on a four-circle Nonius Kappa diffractometer (Enraf-Nonius B.V., Rotterdam, The Netherlands) equipped with a CCD area detector employing graphite monochromated Mo-K*a* radiation (λ = 0.071069 nm) at 300 K. The orientation matrix and unit cell parameters were derived using the program DENZO [51]. No absorption corrections were necessary because of the rather regular crystal shapes and the small dimensions of the investigated specimens. The structures were solved by direct methods and refined with the Oscail program. A quantitative analysis of the structural details was carried out with the program SHELXS-97 [52].

A large piece was cut from the largest crystal (the L sample, see below) for the electrical resistivity and Seebeck coefficient measurements with a ZEM-3 (ULVAC-Riko, Kanagawa, Japan) in the temperature range between room temperature and 850 K, within 2% measurement uncertainties. For these measurements we cut a cuboid of ~7 × 1 × 1 mm^3^ from the original sample.

## 3. Results and Discussion

### 3.1. Chemical Properties

**Figure 2:**
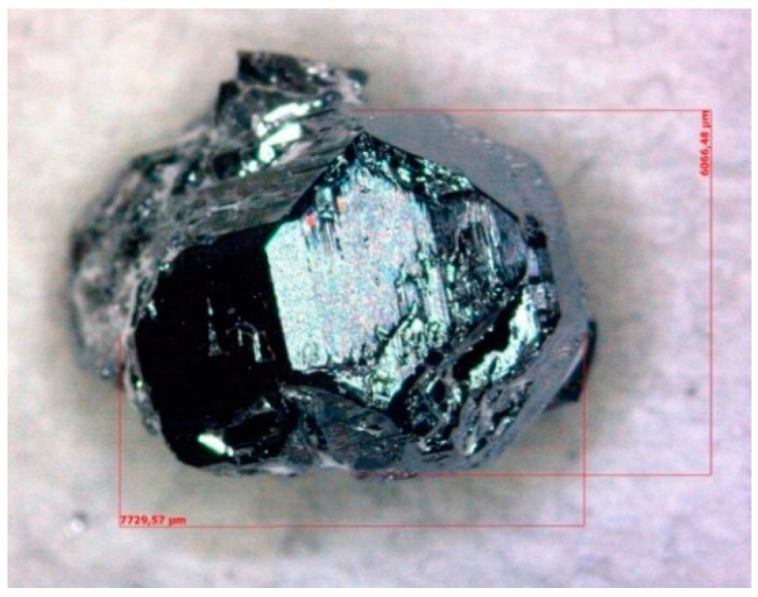
The morphological image of the largest crystal.

Several polyhedral crystals were obtained. The largest one has a size of 7.7 × 6.0 × 5.5 mm^3^ (Figure 2). The crystals are very brittle and inclusions of pure Ga can be found in some crystals. Consequently, it is difficult to cut a big enough sample without inclusion for thermal conductivity measurements (the required size: cylinders with 6 mm in diameter and 1 mm in thickness).

**Figure 3:**
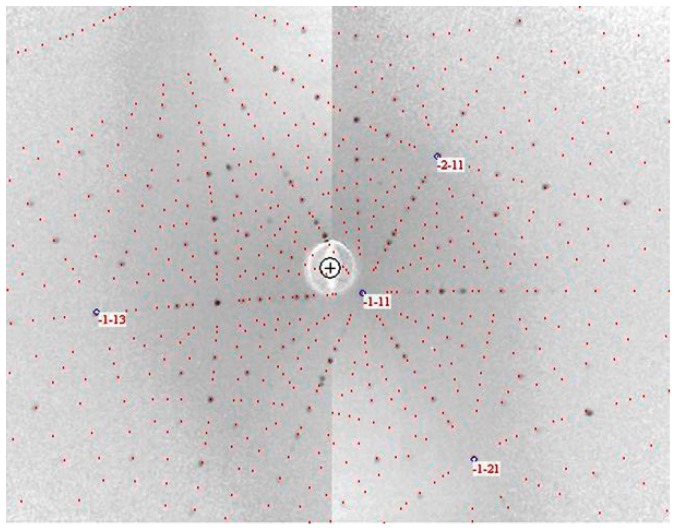
Laue X-ray image for the largest crystal L. The grey points are observed, the red points are simulated for the cubic system (SG: *Pm*-3*n*; a = 1.05324(1) nm). Measurements have been performed from different surfaces.

**Figure 4:**
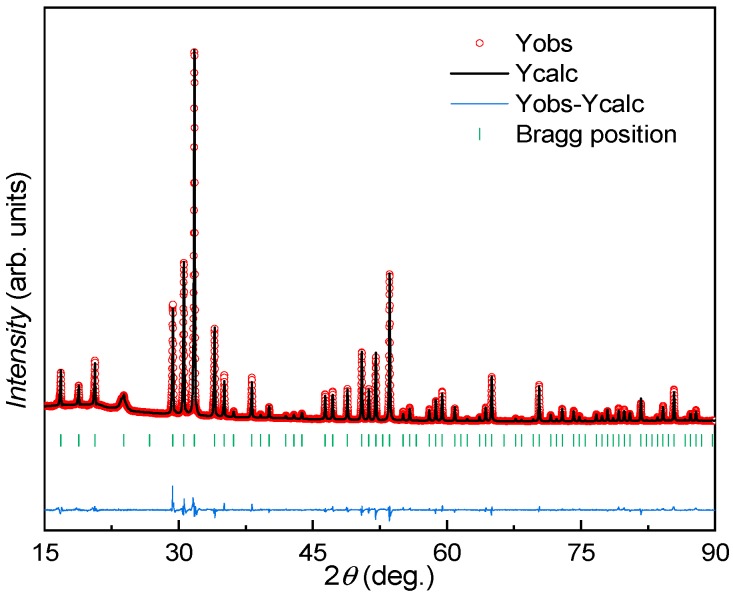
Rietveld refinement of the X-ray powder diffraction (XRD) data for the large crystal L, based on the structure model given in Table 1 (R_F_ = 4,4%, R_B_ = 4.6%).

Figure 3 shows the Laue X-ray image of the largest crystal. The calculated pattern (red points in Figure 3) based on the space group *Pm*-3*n* and the lattice parameter *a* = 1.05324(1) nm can fit well the observed pattern, indicating the high quality of the single crystal. No twin pattern can be seen from different measuring points of the single crystal. The high quality was also confirmed by narrow peaks from X-ray powder diffraction (Figure 4), and by very similar compositions measured by EDX for different parts.

**Figure 5:**
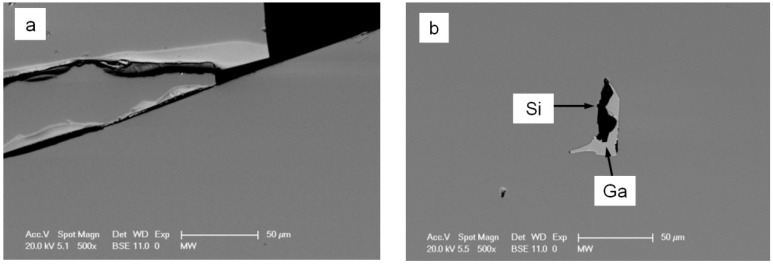
Scanning electron microscope (SEM) images for samples (**a**) L (left) and (**b**) S (right). The main phase (grey color) in both samples is clathrate; the light grey phase in the S sample (right) is Ga (inclusion) and the black phase is Si.

The composition measurements by EDX were focused on two crystals: the one mentioned above with the largest size (denoted by L), and another one with a relatively small size (S) which is used to confirm the similarity of the chemical and physical properties in these crystals. Figure 5 shows the SEM images for these two crystals. We can see cracks (created by polishing because of the brittleness) and inclusions in the L sample, and pure Si and inclusions (mainly Ga) in the S sample. The measured compositions are Ba_14.4(3)_Cu_0.4(1)_Si_59.3(5)_Ga_25.9(5)_ (Ba_8_Cu_0.2_Si_32_Ga_13.8_) for the L sample and Ba_14.3(4)_Cu_0.6(1)_Si_59.5(5)_Ga_25.6(9)_ (Ba_8_Cu_0.3_Si_32_Ga_13.7_) for the S sample, respectively (the compositions in the brackets are normalized by assuming no vacancies in the structure). Both crystals are chemically identical. 

Obviously, the Cu content in both compositions is very small, which is unexpected. The Ga content is around 13.8 at./f.u., which is less than 16 at./f.u. expected from the Zintl rule, also less than 15 at./f.u. in the spark plasma sintered sample [46]. The low Cu content in the crystals implies that the Ga flux would prevent Cu from entering the framework. Cu prefers to form other more stable phases (crystals) with Ga (unfortunately, we did not analyze the phase types of very small crystals, which could be Cu-Ga containing phases). Using other elements such as Sn or Bi as flux might increase the Cu content.

### 3.2. Structural Analysis of Single Crystals

To figure out the Ga preferences in the crystal structure of Ga-substituted IVA clathrates, Si-based clathrates are more suitable for such a purpose than Ge-based ones since the electron difference is larger between Si and Ga than that between Ge and Ga. We selected several single crystals from both L and S samples and collected intensity data for two good crystals. Cu will not influence the analysis of Ga site preferences during the Rietveld refinements of the single crystals X-ray diffraction data since (1) Cu has an X-ray scattering factor similar to Ga, and (2) the amount is very small. Consequently, in our analysis, we excluded the Cu element at the beginning for the occupation study.

The analysis of X-ray data gave the expected crystal structure of type I clathrate (space group: *Pm*-3*n*) with the heavier Ba atoms located at the 2*a* and 6*d* sites and the other elements forming the framework 6*c*, 16*i*, and 24*k* sites. To analyze the distributions of Ga and Si (as mentioned, we ignored Cu in the first stage) in the framework, we applied criteria as (1) the thermal parameters at each site should be physically reasonable under defined occupations; (2) the refined composition should be similar to the one measured by EDX; and (3) obtaining “real” low residual values (R values) from the refinements. Based on these criteria the refinements delivered the structural parameters as shown in Table 1: Ga and Si atoms share all framework sites in different ratios, consistent with the results in the literature for Si- and Ge-based Ga containing type I clathrates [31,35,38,39,42,46]. The refined compositions Ba_8_Si_32.1_Ga_14.2(2)_ for the L sample and Ba_8_Si_32.1_Ga_14.2(2)_ for the S sample are very close to the EDX compositions Ba_8_Cu_0.2_Si_32_Ga_13.8_ and Ba_8_Cu_0.3_Si_32_Ga_13.7_, respectively. The R values are convincingly small and the residual electron densities (the highest peak and deepest hole are 0.61 and −0.82 e^−^/Å^3^ for L, and 0.81 and −0.94 e^−^/Å^3^ for S) are also very sparse, indicating the adequacy of the structure models. From Table 1, we note that the structural parameters, especially the atom occupations of the two crystals, are practically identical, indicating a very good reproducibility of the crystal structures of our crystals.

We also refined the powder diffraction data with the model in which Ba atoms occupy the 2*a* and 6*d* sites and Ga and Si atoms share all framework sites. The refined X-ray pattern is shown in Figure 4. Similar structural parameters have been derived from the Rietveld refinements (not shown here). 

**Figure 6:**
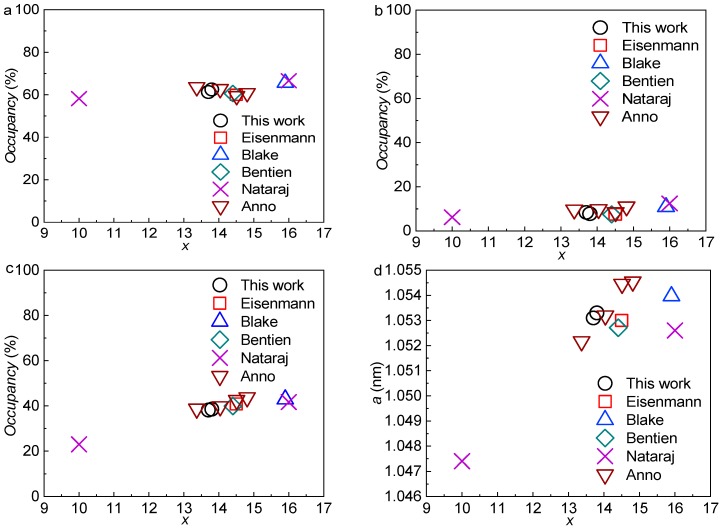
Occupations of Ga at different sites vs the Ga content x in the type I clathrates Ba_8_Ga_x_Si_46−x_: (**a**) at the 6*c* site; (**b**) at the 16*i* site; and (**c**) at the 24*k* site. (**d**) Lattice parameter vs. x. The data from the present work are denoted by black circles.

**Figure 7:**
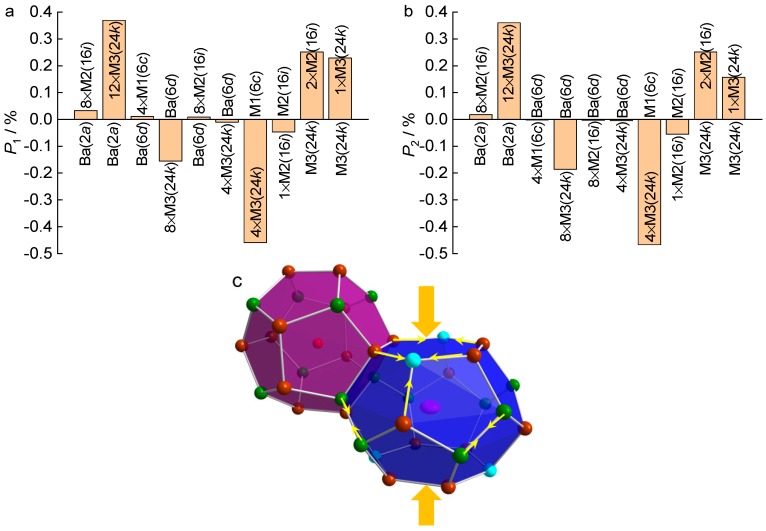
Interatomic distance changes (percentages *P_i_*, definition see Table 2) caused by Cu substitution (**a**) *P*_1_, (**b**) *P*_2_, and (**c**) a sketch shows the changes in the large cage.

Next, we compared our structural parameters with the literature data and revisited the arguments for the site preferences of the Ga atoms in the crystal structure. The comparisons were mainly focused on Ga occupations at different sites, lattice parameters, as well as interatomic distances between relevant atoms (Figure 1). The results are illustrated in Figure 6 and Figure 7.

We were interested in the Ga site preferences in the crystal structure because this has significant influence on both the structural stability and the thermoelectric properties [34]. As predicted from theoretical calculations [34], Ga atoms preferably occupy the 6*c* and 24*k* sites and try to avoid the 16*i* site because it is energetically unfavorable to form bonds between Ga atoms in the framework [35]. An alternative explanation can be that atoms at the 6*c* and 24*k* sites bond together to build distorted tetrahedra, i.e., bond angles deviating from 109.47° for the diamond structure [39], which leads to less energy consumption when Ga locates in these sites than in the 16*i* site [34]. Generally, all the literature data show that the occupation percentages of Ga are around 60.5%, 8.5%, and 37.5% at the 6*c*, 16*i*, and 24*k* sites, respectively, which are almost independent on the composition except for that at the 24*k* site, where a slight increase with increasing Si content can be seen (Figure 6a–c). Christensen et al. [53] even predicted the highest solid solubility of Ga in the Si-based clathrate by the Ga site preference rule, which can be less than 15 at./f.u. This value can be roughly confirmed by the change of lattice parameters with Si content, as shown in Figure 6d. The increase of the lattice parameter with Ga content is due to the replacement of the smaller Si atoms by the Ga atoms. The Ga occupations from our work follow well the literature data.

Interestingly, the Ga content in our single crystals is only around 14 at./f.u., lower than the value 15 at./f.u. predicted earlier. It is likely that the small amount of Cu in the framework reduces the Ga content in order to fulfill the Zintl concept. According to this concept, Cu atoms consume 3 electrons and Ga only 1 electron to form the same electronic configuration as a Si atom in the three-dimensional net structure (diamond-like structure). Based on this picture, 0.3 at./f.u. Cu (in the S sample) equals 0.9 at./f.u. Ga in the ability of accepting electrons. Therefore, from the Zintl concept point of view, the composition of the S sample is analogous to Ba_8_Si_32_Ga_(13.7+0.9)_, in which the Ga content approaches the predicted 15 at./f.u. [53].

Although the Cu content is very small, it causes subtle changes in the crystal structure, which is observed by comparing the interatomic distances with those of Cu-free compounds in which the Si content is similar (~32 at./f.u.). These subtle changes can be used to explain the observed transport properties in our crystals (see below). We compared our data with those from Ref. [46] and the results are shown in Table 2 and Figure 7. The interatomic distances in the samples from Ref. [46] were calculated based on their provided structural data. We can see a shorter interatomic distance between M1(6*c*) and M3(24*k*) and longer ones between M2(16*i*) and M3(24*k*), as well as M3(24*k*) and M3(24*k*) in our crystals than the Cu-free samples, reflected by the negative values for the former and positive values for the latter ones in *P*_1_ and *P*_2_ in Figure 7. The relatively higher absolute values of *P_i_* (*i* = 1, 2) for M1(6*c*)-M3(24*k*) than the other two are due to more Si occupying the 6*c* site caused by Cu substitution for Ga. This may also be an indication that Cu locates at the 6*c* site, as normally seen from transition elements in other type I clathrates [11,12,13,14,15,16,17,18,19,20,21,22,23,24,25,26]. As a consequence of these distance changes, the shape of the two cages has also changed, which is reflected by positive values for Ba(2*a*)-M3(24*k*) and negative values for Ba(6*d*)-M3(24*k*), as well as minor values for the other interatomic distances (e.g., Ba(6*d*)-M2(16*i*)) in *P_i_* (*i* = 1, 2) in Figure 7. The atomic bonds in the six-atom-ring of the large cage are shortened and, simultaneously the cage is compressed along the direction perpendicular to the six-atom-ring plane (Figure 7c), which could lead to a high efficiency on the electron transfer from Ba atoms to the framework atoms [35,54,55,56,57]. This is evident from a higher electron concentration in our crystals than in the Cu-free samples as shown below. 

The changes in interatomic distance caused by Cu substitution is not directly from the size difference between Cu and Ga atoms (actually, in covalent state, the atomic radii of Cu and Ga are similar), but from the substitution reducing the Ga content and simultaneously increasing the Si content, which decreases the involved bonds discussed above. Meanwhile, the electronic balance is maintained according to the Zintl rule. 

### 3.3. Thermoelectric Properties

**Figure 8:**
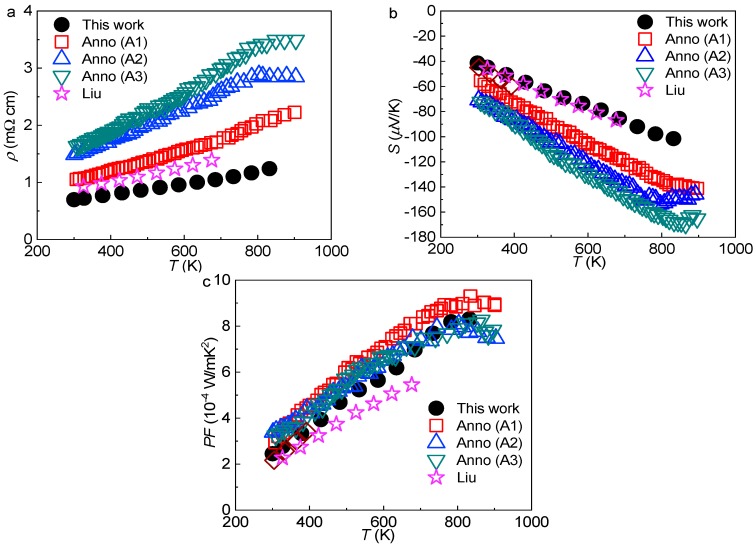
Temperature dependent (**a**) electrical resistivity *ρ*, (**b**) Seebeck coefficient *S*, and (**c**) power factor *PF* for the L sample. Comparisons are made with the data from different sources: open red squares represent Ba_7.65_Ga_14.51_Si_31.84_ from Ref. [46]; open blue triangles represent Ba_7.60_Ga_14.78_Si_31.63_ from Ref. [46]; open green inverted triangles represent Ba_7.79_Ga_14.81_Si_31.40_ from Ref. [46]; purple stars represent Ba_7.5_Cu_0.8_Si_30.7_Ga_14.4_ from Ref. [50].

Temperature dependent electrical resistivity *ρ*(*T*), Seebeck coefficient *S*(*T*), and power factor *PF*(*T*) are shown in Figure 8. The literature data for samples of a similar Si content are included for comparison. For all compounds, the electrical resistivity exhibits metallic-like behavior, and the Seebeck coefficient is negative, which indicates that electrons are the dominating charge carriers, well in coincidence with the Zintl prediction.

According to the Zintl counting scheme, we estimated the charge carrier concentrations for all selected compounds by *n*_1_ = 2*x*_Ba_ − 3*y*_Cu_ − 1*z*_Ga_, where *x*_Ba_, *y*_Cu_, and *z*_Ga_ are the content for Ba, Cu, and Ga in at./f.u., respectively. The prefactor is the electron number (valence electrons) that the corresponding atom can provide or accept. Here we fixed the Ba content *x*_Ba_ as 8 at./f.u. (both cages are assumed to be fully filled) though the EDX content could be less in some samples. The results are given in Table 3. We also estimated the charge carrier concentration by the temperature dependent Seebeck coefficient *S*(*T*) using the equation:

(1) $$S=\frac{2\pi^{2}k_{B}^{2}m_{e}}{e\hbar^{2}{\left(3n\pi^{2}\right)}^{2/3}}T,$$

where *n* is the charge carrier concentration, *m_e_* the free electron mass, *e* the charge, and the others have the general meaning. Since the Debye temperature is higher than 300 K in this type of clathrates [46], the estimate of *n* with the linear relationship at the high temperature range (300–~800 K) is justified. The charge carrier concentration derived from Equation (1) was labeled *n*_2_ in Table 3. The difference between *n*_1_ and *n*_2_ can be from the underestimated effective charge carrier mass, or the inaccuracies of the EDX compositions.

By comparing the charge carrier concentration in our crystal with the Cu-free compounds, we noticed that although the predicted *n*_1_ in the L sample is very close to the A1 and A2 samples, *n*_2_ in the former sample is larger than in the latter ones. This explains that the Seebeck coefficient (absolute value) is lower in the L sample than in the A1 and A2 samples (Figure 8b), and also partially explains the lower electrical resistivity in Figure 8a (another reason can be the lack of the grain boundary scattering for electrons in the single crystal sample). The higher *n*_2_ might arise from the changes of the crystal structure caused by Cu substitution, i.e., the compression in the direction perpendicular to the six-atom-ring plane of the large cage (Figure 7c) increases the electron transfer efficiency, as it enhances the wave function overlap between the guest and framework atoms [58].

Combining the electrical resistivity and Seebeck coefficient, we show the power factor *PF* in Figure 8c. At relatively low temperatures, the *PF* from our work is lower than for the Cu-free samples, which is due to the high charge carrier concentration. At higher temperatures, the *PF* of the single crystal with Cu substitution is comparable to the Cu-free polycrystalline samples. In our crystal, the highest *PF* value reached is 0.83 mW/mK^2^ at 832 K. 

## 4. Conclusions

In summary, we have synthesized single crystals of type I clathrate by a self-flux method in the Ba-Cu-Si-Ga quaternary system. The crystals have been characterized by X-ray powder diffraction, Laue measurements, X-ray single crystal diffraction, EDX measurements, and transport property measurements. We proved that we can obtain quaternary type I clathrates in this system, but the Cu content is very small. Even so, the small amount of Cu affects the crystal structure and the transport properties. Structural investigation showed that the site preferences of Ga generally follow the rule of sharing the 6*c* and 24*k* sites with the IVA elements and avoiding the 16*i* site to prevent Ga-Ga direct bonds. By analyzing the bonds (interatomic distances), we found that the Cu substitution compressed the large cage along the direction perpendicular to the six-atom-ring plane, which could enhance the capability/efficiency of electron transfer from the Ba atoms enlarging the wave function overlap between the guest and framework atoms. A higher charge carrier concentration has been observed in the Cu-containing single crystal, which reduces both the electrical resistivity and Seebeck coefficient. We attributed the structural and electronic changes to the Cu substitution (1) increasing the Si/Ga ratio by electron balance in this Zintl compound and (2) shortening bonds in certain directions because of the Ga site preferences, rather than a simply direct size effect of Cu replacing Ga. We thus expect that this could be a strategy for Zintl compounds for tuning electronic parameters to reach high thermoelectric performance.

## Figures and Tables

**Table 1:** Structural data for the two single crystals (L and S) with approximate compositions Ba_8_Si_32.1_Ga_14.2(2)_ and Ba_8_Si_32.1_Ga_14.2(2)_ derived from X-ray single crystal refinements. Data collection: 2 ≤ 2θ ≤ 72.5; 75 s/frame; Total number of frames: 210 frames in 5 sets; Mosaicity: <0.43; Derived space group (SG): *Pm*-3*n*.

Parameters/Notes	L	S
Formula from refinement	Ba_8_Si_32.1_Ga_14.2(2)_	Ba_8_Si_32.1_Ga_14.2(2)_
Composition (EDX, at/u.c.)	Ba_8_Cu_0.2_Si_32_Ga_13.8_	Ba_8_Cu_0.3_Si_32_Ga_13.7_
*a* (nm)	1.05332(20)	1.05317(20)
*μ*_abs_ (mm^−1^)	16.32	15.25
Reflections in refinement	513(F_o_) ≥ 4σ (F_o_)	497 (F_o_) ≥ 4σ(F_o_)
Number of variables	22	22
R_F_^2^ = ∑|F_o_^2^ − F_c_^2^|/∑F_o_^2^	0.0097	0.0132
R_int_	0.0351	0.0473
wR2	0.0244	0.0270
GOF	1.618	1.338
Extinction coefficient	0.0078(2)	0.0056(2)
R*_e_* (highest peak; deepest hole) (e^−^/Å^3^)	0.61; −0.82	0.81; −0.94
Ba at 2a (0,0,0) U*_eq_* 10^2^ (nm^2^)	0.00948(6)	0.00975(7)
U*_ii_*, *i* = 1; 2; 3	0.00948(6)	0.00975(7)
Ba at 6d (1/4, 1/2, 0) U*_eq_*	0.02670(6)	0.02690(7)
U_11_; U*_ii_*, *i* = 2; 3	0.01385(8); 0.03312(8)	0.01407(10); 0.03331(9)
M1 at 6*c* (1/4, 0, 1/2), Occ.	3.7(0)Ga+2.3(1)Si	3.7(0)Ga+2.3(1)Si
U*_eq_*	0.00861(10)	0.00863(12)
U_11_; U*_ii_*, *i* = 2; 3	0.00946(15); 0.00819(11)	0.00935(19); 0.00827(14)
M2 at 16*i* (*x*, *x*, *x*), Occ.	1.2(1)Ga+14.8(2)Si	1.3(1)Ga+14.7(2)Si
U*_eq_*	0.00836(10)	0.00839(12)
*x*	0.18540(2)	0.18540(2)
U*_ii_*, *i* = 1;2;3; U_23_ = U_13_ = U_12_	0.00836(10); −0.00097(7)	0.00839(12); −0.00087(8)
M3 at 24*k* (0, *y*, *z*), Occ.	9.3(1)Ga+15.0(3)Si	9.2(1)Ga+15.1(4)Si
U*_eq_*	0.00822(6)	0.00840(7)
*y*, *z*	0.30621(2); 0.11932(2)	0.30626(2); 0.11925(2)
U_11_; U_22_	0.00843(10); 0.00758(10)	0.00862(12); 0.00777(12)
U_33_; U_23_	0.00865(10); −0.00033(6)	0.00882(12); −0.00033(8)

**Table 2:** Selected refined interatomic distances (Å) for L and S and for the samples from Ref. [46] (A1 and A2). For comparison, we calculated the change percentages (*P*_i_ (%)) based on the Cu-free sample A1: *P*_1_ = (*d*_L_ − *d*_A1_)/*d*_A1_; *P*_2_ = (*d*_S_ − *d*_A1_)/*d*_A1_. The interatomic distances of A1 and A2 were calculated by Bond Str with structural data. We show here A2 for comparison with the Cu-free samples.

Notes and References		L	S	Anno (A1)	Anno (A2)	*P*_1_	*P*_2_
Formula from refinement		Ba_8_Si_32.1(6)_Ga_14.2(2)_	Ba_8_Si_32.1(7)_Ga_14.2(2)_	Ba_8_Si_31.1_Ga_14.9_	Ba_8_Si_30.9_Ga_15.1_		
Composition (at/u.c.)		Ba_8_Cu_0.2_Si_32_Ga_13.8_	Ba_8_Cu_0.3_Si_32_Ga_13.7_	Ba_7.71_Si_32.24_Ga_14.04_	Ba_8_Si_31.84_Ga_14.51_		
Ba(2*a*)	−8M2(16*i*)	3.3824(2)	3.3819(1)	3.3813(1)	3.3883(1)	0.033	0.018
	−12M3(24*k*)	3.4616(1)	3.4613(1)	3.4489(3)	3.4594(3)	0.369	0.360
Ba(6*d*)	−4M1(6*c*)	3.7240(2)	3.7235(4)	3.7236(3)	3.7280(2)	0.011	−0.002
	−8M3(24*k*)	3.5610(2)	3.5599(3)	3.5665(2)	3.5680(2)	−0.155	−0.186
	−8M2(16*i*)	3.9062(3)	3.9057(2)	3.9058(2)	3.9096(3)	0.009	−0.003
	−4M3(24*k*)	4.0533(4)	4.0535(3)	4.0537(4)	4.0575(4)	−0.010	−0.005
M1(6*c*)	−4M3(24*k*)	2.4619(2)	2.4617(2)	2.4732(2)	2.4700(1)	−0.459	−0.467
M2(16*i*)	−1M2(16*i*)	2.3572(1)	2.3570(1)	2.3583(1)	2.3553(1)	−0.047	−0.055
M3(24*k*)	−2M2(16*i*)	2.4326(0)	2.4326(1)	2.4265(1)	2.4330(1)	0.251	0.251
	−1M3(24*k*)	2.5136(2)	2.5118(2)	2.5079(1)	2.5149(2)	0.229	0.157

**Table 3:** Ga and Cu contents (*y*_Ga_, *z*_Cu_) in samples for comparisons, which are used for estimation of the charge carrier concentration *n*_1_ by the Zintl rule; *n*_2_ is derived from Equation (1) (see text).

Sample Code	*y*_Ga_	*z*_Cu_	*a*	*n*_1_	*n*_2_
	at./f.u.	at./f.u.	nm	1/f.u.	1/f.u.
**L**	13.8	0.2	1.05332(20)	1.6	1.10
**A1**	14.04	-	1.053189	1.96	0.69
**A2**	14.51	-	1.054450	1.49	0.60
**A3**	14.81	-	1.054540	1.19	0.51
**Liu**	14.7(6)	0.7(9)	1.05294(1)	−0.8 ^1^	1.10

^1^ The negative value indicates that the carrier type is hole-like, which is in contrast to *n*_2_ and the *S* sign. We thus eliminated this sample for the comparison in the text.

## References

[1] Wood C. (1988). Materials for thermoelectric energy conversion. Rep. Prog. Phys..

[2] Nolas G.S., Cohn J.L., Slack G.A., Schujman S.B. (1998). Semiconducting Ge clathrates: Promising candidates for thermoelectric applications. Appl. Phys. Lett..

[3] Goldsmid H.J., Rowe D.M. (1995). Conversion Efficiency and Figure-of-Merit. CRC Handbook of Thermoelectrics.

[4] Slack G.A., Rowe D.M. (1995). New Materials and Performance Limits for Thermoelectric Cooling. CRC Handbook of Thermoelectrics.

[5] Dolyniuk J., Owens-Baird B., Wang J., Zaikina J.V., Kovnir K. (2016). Clathrate thermoelectrics. Mater. Sci. Eng. R Rep..

[6] Dong J., Sankey O.F., Ramachandran G.K., McMillan P.F. (2000). Chemical trends of the rattling phonon modes in alloyed germanium clathrates. J. Appl. Phys..

[7] Dong J., Sankey O.F., Myles C.W. (2001). Theoretical study of the lattice thermal conductivity in Ge framework semiconductors. Phys. Rev. Lett..

[8] Shevelkov A.V., Kovnir K., Fässler T.F. (2011). Zintl clathrates. Zintl Phases: Principles and Recent Developments.

[9] Shevelkov A.V., Nikitin M., Skipidarov S. (2016). Thermoelectric Power Generation by Clathrates. Thermoelectrics for Power Generation—A Look at Trends in the Technology.

[10] Rogl P., Rowe D.M. (2006). Formation and Crystal Chemistry of Clathrates. Thermoelectrics Handbook: Macro to Nano.

[11] Yan X., Ikeda M., Zhang L., Bauer E., Rogl P., Giester G., Prokofiev A., Paschen S. (2018). Suppression of vacancies boosts thermoelectric performance in type-I clathrates. J. Mater. Chem. A.

[12] Cordier G., Woll P. (1991). Neue ternäre intermetallische Verbindungen mit Clathratstruktur: Ba_8_(T,Si)_6_Si_40_ und Ba_6_(T,Ge)_6_Ge_40_ mit T≡Ni,Pd,Pt,Cu,Ag,Au. J. Less-Common. Met..

[13] Li Y., Chi J., Gou W., Khandekar S., Ross J.H. (2003). Structure and stability of Ba–Cu–Ge type-I clathrates. J. Phys. Condens. Mater..

[14] Anno H., Hokazono M., Kawamura M., Matsubara K. Effect of transition element substitution on thermoelectric properties of semiconductor clathrate compounds. Proceedings of the 22nd International Conference on Thermoelectrics.

[15] Anno H., Hokazono M., Takakura H., Matsubara K. Thermoelectric Properties of Ba_8_Au_x_Ge_46−x_ Clathrate Compounds. Proceedings of the 2005 International Conference on Thermoelectrics.

[16] Hokazono M., Anno H., Matsubara K. (2005). Effect of Cu Substitution on Thermoelectric Properties of Ge Clathrates. Mater. Trans..

[17] Johnsen S., Bentien A., Madsen G.K.H., Iversen B.B., Nygren M. (2006). Crystal Structure, Band Structure, and Physical Properties of Ba_8_Cu_6−x_Ge_40+x_ (0 ≤ x ≤ 0.7). Chem. Mater..

[18] Johnsen S., Bentien A., Madsen G.K.H., Nygren M., Iversen B.B. Copper containing germanium clathrates. Proceedings of the 2005 International Conference on Thermoelecrics.

[19] Johnsen S., Bentien A., Madsen G.K.H., Nygren M., Bo B.I. (2007). Crystal structure and transport properties of nickel containing germanium clathrates. Phys. Rev. B.

[20] Melnychenko-Koblyuk N., Grytsiv A., Rogl P., Rotter M., Bauer E., Durand G., Kaldarar H., Lackner R., Michor H., Royanian E. (2007). Clathrate formation in the Ba-Pd-Ge system: Phase equilibria, crystal structure, and physical properties. Phys. Rev. B.

[21] Melnychenko-Koblyuk N., Grytsiv A., Rogl P., Rotter M., Lackner R., Bauer E., Fornasari L., Marabelli F., Giester G. (2007). Structure and physical properties of type-I clathrate solid-solution Ba_8_Pt_x_Ge_46_−x−y□y (□ = vacancy). Phys. Rev. B.

[22] Melnychenko-Koblyuk N., Grytsiv A., Berger S., Kaldarar H., Michor H., Röhrbacher F., Royanian E., Bauer E., Rogl P., Schmid H. (2007). Ternary clathrates Ba–Cd–Ge: Phase equilibria, crystal chemistry and physical properties. J. Phys. Condens. Mater..

[23] Melnychenko-Koblyuk N., Grytsiv A., Fornasari L., Kaldarar H., Michor H., Röhrbacher F., Koza M., Royanian E., Bauer E., Rogl P. (2007). Ternary clathrates Ba–Zn–Ge: Phase equilibria, crystal chemistry and physical properties. J. Phys. Condens. Mater..

[24] Yan X., Giester G., Bauer E., Rogl P., Paschen S. (2010). Ba-Cu-Si Clathrates: Phase Equilibria and Crystal Chemistry. J. Electron. Mater..

[25] Nasir N., Grytsiv A., Melnychenko-Koblyuk N., Rogl P., Bednar I., Bauer E. (2010). Crystal structure and physical properties of quaternary clathrates Ba_8_Zn_x_Ge_46−x−y_Si_y_, Ba_8_(Zn,Cu)_x_Ge_46−x_ and Ba_8_(Zn,Pd)_x_Ge_46−x_. J. Solid State Chem..

[26] Xu J., Wu J., Shao H., Heguri S., Tanabe Y., Liu Y., Liu G., Jiang J., Jiang H., Tanigaki K. (2015). Structure and thermoelectric properties of the n-type clathrate Ba_8_Cu_5.1_Ge_40.2_Sn_0.7_. J. Mater. Chem. A.

[27] Liang Y., Böhme B., Reibold M., Schnelle W., Schwarz U., Baitinger M., Lichte H., Grin Y. (2011). Synthesis of the Clathrate-I Phase Ba_8−x_Si_46_ via Redox Reactions. Inorg. Chem..

[28] Liang Y., Böhme B., Vasylechko L., Baitinger M., Grin Y. (2013). In-situ investigation of the thermal decomposition of clathrate-I Ba_6.2_Si_46_. J. Phys. Chem. Solids.

[29] Castillo R., Schnelle W., Bobnar M., Burkhardt U., Böhme B., Baitinger M., Schwarz U., Grin Y. (2015). The Clathrate Ba_8−x_Si_46_ Revisited: Preparation Routes, Electrical and Thermal Transport Properties. Z. Anorg. Allg. Chem..

[30] Saramat A., Svensson G., Palmqvist A.E.C., Stiewe C., Mueller E., Platzek D., Williams S.G.K., Rowe D.M., Bryan J.D., Stucky G.D. (2006). Large thermoelectric figure of merit at high temperature in Czochralski-grown clathrate Ba_8_Ga_16_Ge_30_. J. Appl. Phys..

[31] Eisenmann B., Schäfer H., Zagler R. (1986). Die verbindungen A^II^_8_B^III^_16_B^IV^_30_ (A^II^ ≡ Sr, Ba; B^III^ ≡ Al, Ga; B^IV^ ≡ Si, Ge, Sn) und ihre käfigstrukturen. J. Less-Common. Met..

[32] Cohn J.L., Nolas G.S., Fessatidis V., Metcalf T.H., Slack G.A. (1999). Glasslike Heat Conduction in High-Mobility Crystalline Semiconductors. Phys. Rev. Lett..

[33] Kuznetsov V.L., Kuznetsova L.A., Kaliazin A.E., Rowe D.M. (2000). Preparation and thermoelectric properties of A^II^_8_B^III^_16_B^IV^_30_ clathrate compounds. J. Appl. Phys..

[34] Blake N.P., Latturner S., Bryan J.D., Stucky G.D., Metiu H. (2001). Band structures and thermoelectric properties of the clathrates Ba_8_Ga_16_Ge_30_, Sr_8_Ga_16_Ge_30_, Ba_8_Ga_16_Si_30_, and Ba_8_In_16_Sn_30_. J. Chem. Phys..

[35] Blake N.P., Bryan D., Latturner S., Møllnitz L., Stucky G.D., Metiu H. (2001). Structure and stability of the clathrates Ba_8_Ga_16_Ge_30_, Sr_8_Ga_16_Ge_30_, Ba_8_Ga_16_Si_30_, and Ba_8_In_16_Sn_30_. J. Chem. Phys..

[36] Mudryk Y., Rogl P., Paul C., Berger S., Bauer E., Hilscher G., Godart C., Noël H. (2002). Thermoelectricity of clathrate I Si and Ge phases. J. Phys. Conders. Mater..

[37] Nataraj D., Nagao J., Ferhat M., Ebinuma T. High temperature thermoelectric properties of Arc-melted Ba_8_M_16_Si_30_ (M = Al, Ga) clathrates. Proceedings of the 21st International Conference on Thermoelectronics.

[38] Nataraj D., Nagao J., Ferhat M., Ebinuma T. (2003). Structure, high temperature transport, and thermal properties of Ba_8_Ga_x_Si_46−x_ (x = 10 and 16) clathrates prepared by the arc melting method. J. Appl. Phys..

[39] Nataraj D., Nagao J. (2004). Structure and Raman scattering study on Ba_8_Ga_x_Si_46−x_ (x = 10 and 16) type I clathrates. J. Solid State Chem..

[40] Qiu L., Swainson I.P., Nolas G.S., White M.A. (2004). Structure, thermal, and transport properties of the clathrates Sr_8_Zn_8_Ge_38_, Sr_8_Ga_16_Ge_30_, and Ba_8_Ga_16_Si_30_. Phys. Rev. B.

[41] Bentien A., Iversen B.B., Bryan J.D., Stucky G.D., Palmqvist A.E.C., Schultz A.J., Henning R.W. (2002). Maximum entropy method analysis of thermal motion and disorder in thermoelectric clathrate Ba_8_Ga_16_Si_30_. J. Appl. Phys..

[42] Bentien A., Nishibori E., Paschen S., Iversen B.B. (2005). Crystal structures, atomic vibration, and disorder of the type-I thermoelectric clathrates Ba_8_Ga_16_Si_30_, Ba_8_Ga_16_Ge_30_, Ba_8_In_16_Ge_30_, and Sr_8_Ga_16_Ge_30_. Phys. Rev. B.

[43] Deng S., Tang X., Tang R. (2009). Synthesis and high temperature thermoelectric transport properties of Si-based type-I clathrates. Chin. Phys. B.

[44] Anno H., Yamada H., Nakabayashi T., Hokazono M., Shirataki R. (2012). Composition dependence of thermoelectric properties in polycrystalline type-I Ba_8_Ga_x_Si_46−x_ (nominal x = 14–18). AIP Conf. Proc..

[45] Anno H., Yamada H., Nakabayashi T., Hokazono M., Shirataki R. (2012). Influence of preparation conditions on thermoelectric properties of Ba_8_Ga_16_Si_30_ clathrate by combining arc melting and spark plasma sintering methods. J. Phys. Conf. Ser..

[46] Anno H., Yamada H., Nakabayashi T., Hokazono M., Shirataki R. (2012). Gallium composition dependence of crystallographic and thermoelectric properties in polycrystalline type-I Ba_8_Ga_x_Si_46−x_ (nominal x = 14–18) clathrates prepared by combining arc melting and spark plasma sintering methods. J. Solid State Chem..

[47] Li F., Liu L., Qiu H., Cao G., Li Y. (2013). Effect of Spark Plasma Sintering on the Crystal Structure and Thermoelectric Properties of Ba_8_Si_30_Ga_16_. Mater. Rev. B.

[48] Deng S., Tang X., Xiong C., Zhang Q. (2007). Synthesis and Electrical Transmission Characteristics of Type-I Ba_8_Ga_16_Zn_x_Si_30−x_ Clathrates. Chin. J. Semicond..

[49] Bi S., Liu L., Li F., Wang Z., Pan H., Li Y. (2015). Thermoelectric property of Ba_8_Ga_14_Cu_2_Si_30_. New Chem. Mater..

[50] Liu L., Song B., Li F., Wang Z., Pan H., Li Y. (2015). Thermoelectric Properties of Ba_8_Ga_15_XSi_30_ (X = Ga, Zn, Cu). J. Inorg. Mater..

[51] Nonius Kappa CCD Program (1998). Package: COLLECT, DEZO, SCALEPACK, SORTAV.

[52] Sheldrick G.M. (1997). Program for Crystal Structure Refinement.

[53] Christensen M., Iversen B.B. (2007). Host Structure Engineering in Thermoelectric Clathrates. Chem. Mater..

[54] Saito S., Oshiyama A. (1995). Electronic structure of Si_46_ and Na_2_Ba_6_Si_46_. Phys. Rev. B.

[55] Bentien A., Palmqvist A.E.C., Bryan J.D., Latturner S., Stucky G.D., Furenlid L., Iversen B.B. (2000). Experimental Charge Densities of Semiconducting Cage Structures Containing Alkaline Earth Guest Atoms. Angew. Chem. Int. Ed..

[56] Moriguchi K., Yonemura M., Shintani A., Yamanaka S. (2000). Electronic structures of Na_8_Si_46_ and Ba_8_Si_46_. Phys. Rev. B.

[57] Kitano A., Moriguchi K., Shintani A., Fukuoka H., Yamanaka S., Nishibori E., Takata M., Sakata M. (2001). Structural Properties and Thermodynamic Stability of Ba-Doped Silicon Type-I Clathrates Synthesized Under High Pressure. Phys. Rev. B.

[58] Nenghabi E.N., Myles C.W. (2008). First-principles calculations of the vibrational and thermal properties of the type-I clathrates Ba_8_Ga_16_Si_x_Ge_30−x_ and Sr_8_Ga_16_Si_x_Ge_30−x_. Phys. Rev. B.

